# Animal welfare knowledge, attitudes, and practices among livestock holders in Ethiopia

**DOI:** 10.3389/fvets.2022.1006505

**Published:** 2022-11-07

**Authors:** Gezahegn Alemayehu, Tsega Berhe, Eyob Gelan, Mulugeta Mokria, Jarso Jaldessa, Jarso Molu, Barbara Wieland, Theodore Knight-Jones, Rebecca E. Doyle

**Affiliations:** ^1^Animal and Human Health, International Livestock Research Institute, Addis Ababa, Ethiopia; ^2^World Agroforestry (ICRAF), ILRI Campus, Addis Ababa, Ethiopia; ^3^Vétérinaires Sans Frontières Suisse, Yabello, Ethiopia; ^4^Institute of Virology and Immunology, Mittelhäusern, Switzerland; ^5^Department of Infectious Diseases and Pathobiology, Vetsuisse Faculty, University of Bern, Bern, Switzerland; ^6^Royal (Dick) School of Veterinary Studies, University of Edinburgh, Edinburgh, United Kingdom

**Keywords:** crop-livestock, item response theory, livelihoods, pastoralist, smallholder farmer

## Abstract

Improving animal welfare is a human responsibility and influenced by a person's values and experiences. Thus, it is critical to have an in-depth understanding of the knowledge, attitudes, and practices (KAP) of animal welfare among animal owners. For livestock in Ethiopia, the greatest proportion of livestock are reared by pastoral and mixed crop-livestock communities. A cross-sectional survey covering a range of species and animal welfare aspects was carried out on a total of 197 household (117 pastoral and 80 crop-livestock owners) and recorded information on 34 animal welfare KAP items. Item response theory models (IRT) were fitted to the data from KAP items to estimate the probability of correctly answering an item. This was used as a function of the respondents' KAP level. Overall, the highest percentage of desirable scores was recorded for the knowledge scale (35.7%) and the lowest was for the practice scale (24.6%). A significant correlation (*P* < 0.01) was found between knowledge of the farmers and their attitude toward animal welfare and self-reported practices. Generally, households practicing mixed crop-livestock farming system had better animal welfare knowledge, attitude, and practice than pastoralist. Mixed crop-livestock farmers had better knowledge on items related to observing the nutrition condition of the animal, animal-human relationship, the importance of water, and health inspection compared to pastoralists. In contrast, pastoralists had better knowledge of items related to natural behavior expression, animal care, and animal suffering than mixed crop-livestock farmers. Pastoralists had 3.3-times higher odds than mixed crop-livestock farmers to have a positive attitude to train their animals without beating. KAP scores demonstrate the need for targeted training to improve animal well-being (i.e., housing, management, nutrition, disease prevention and treatment, responsible care, humane handling) across livestock holding communities in Ethiopia.

## Introduction

In Ethiopia, smallholder farmers depend on livestock for food, income, and other socio-economic benefits (1). Most livestock production in this setting can be classified as low input and is largely extensive. Improved animal welfare in this context is strongly linked to farm productivity, food security, and human health (2–4). However, the welfare of livestock managed under these farming systems can be poor as a result of several factors including limited resources, inadequate knowledge and skills of animal keepers, and weak veterinary services (5–7). This subsequently limits the potential contribution of livestock sectors toward food and nutritional security and improved livelihoods, both at a household level and to the national economy (8). Moreover, the health of animals and the safety of animal products are compromised due to the burden of infectious diseases and the frequent use of antibiotics (9, 10).

Livestock owners are responsible for ensuring all aspects of animal welfare, including proper management, housing, nutrition, disease prevention and treatment, animal care, human handling, and when necessary, humane killing (11). Livestock owners in Ethiopia mostly describe animal welfare as related to the biological needs of the animals but do also recognize their animals' affective state and behavioral needs (12, 13). It is not clear their knowledge of different components of animal welfare, however, nor how well they are putting these into practice.

Overall animal welfare in Ethiopia faces numerous challenges that have not been addressed. Thus, understanding welfare knowledge, attitude, and practice (KAP) among livestock keepers is an important step toward identifying the gaps in animal care and providing a proper recommendation that will help to improve animal welfare and well-being (14, 15). It is also important to assess the association between the probability of a correct response and the characteristics of the measurement tool. Methods based on Item Response Theory (IRT) provide an important description of each item (question) in the form of item parameter estimates such as difficulty and discrimination, and KAP score (16).

Here we present a novel tool to assess KAP around animal welfare amongst smallholder farming communities in Ethiopia. Understanding how animal welfare KAP items function differently in relation to certain factors is also important to develop effective community training initiatives and policy directions. In the case of this study, we aimed to understand if the factors of farming practice, gender, and environmental differences were influencing animal welfare KAP.

## Methods

### Study design and setting

**Figure 1:**
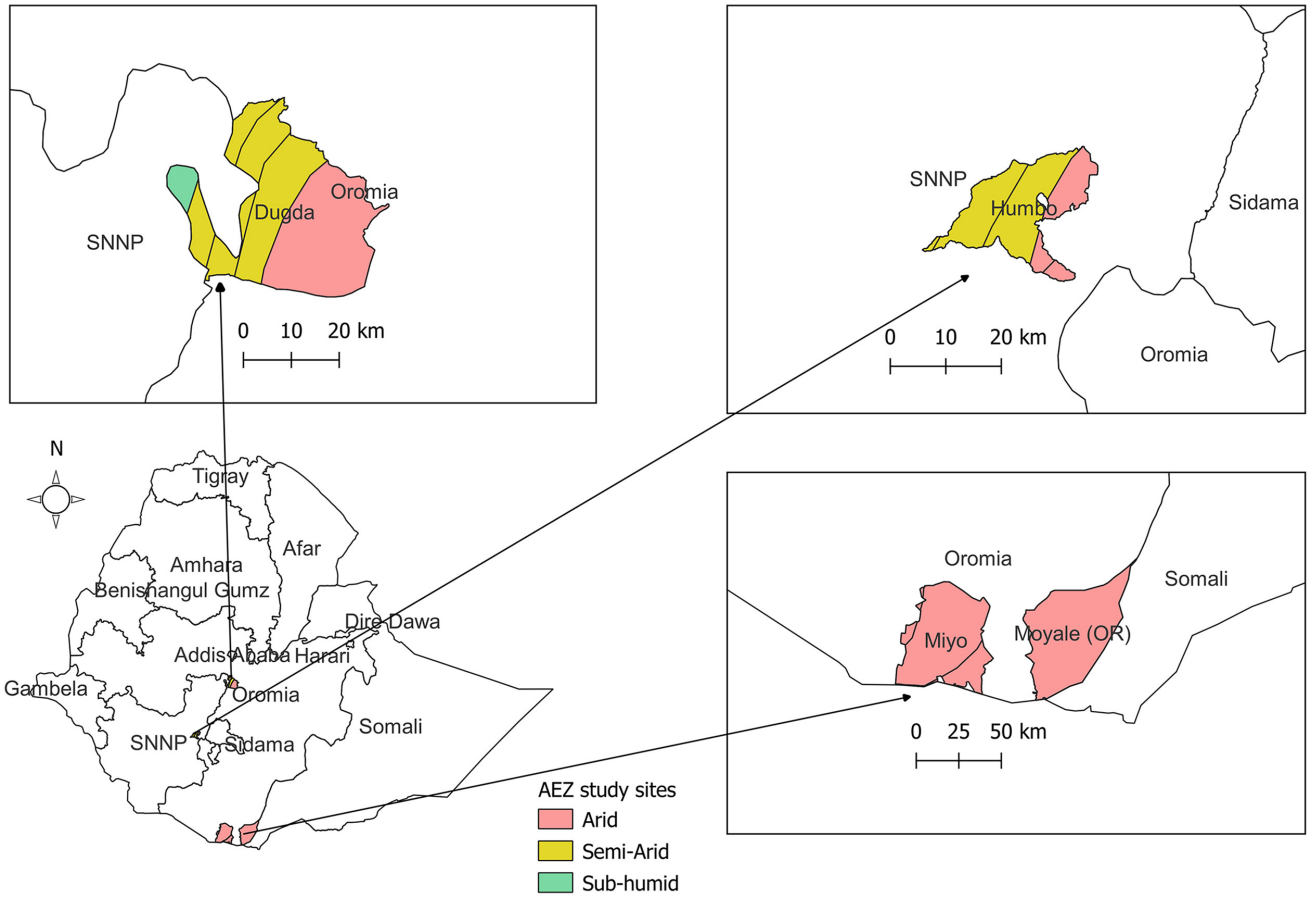
The location of the study areas in Ethiopia. Humbo in Southern Nation Nationality and People (SNNP) regional state and Dugda, Moyale and Miyo in Oromia regional state. Gray line indicates regional boundaries of Ethiopia. AEZ, agroecolog zone.

A cross-sectional study was conducted from February to August 2021 in four purposefully selected districts in two regional states of Ethiopia. Humbo was selected from Southern Nation Nationality and People (SNNP) regional state; Dugda, Moyale, and Miyo were selected from Oromia regional state (Figure 1). Tree coverage differs throughout the districts. In each district, two kebeles (which are the smallest administrative unit in Ethiopia) with relatively good tree access or relatively limited tree access areas were purposively targeted for data collection.

Humbo and Dugda districts represent the mixed crop-livestock production system and were selected for this study based on their potential for agroforestry farming. Humbo and Dugda districts have a total population of 125,000 (50% female) and 145,000 (49% female), respectively (17). In both districts, rural livelihood mainly depends on a mixed crop-livestock farming system in which farmers produce crops, for household consumption and sale and rear livestock simultaneously. Dugda has three agro-climatic zones: arid, semi-arid, and sub-humid. Whereas, Humbo has two agro-climatic zones: arid and semi-arid agro-climatic zones (18).

Moyale and Miyo districts of the Borana zone represent the traditional lowland pastoral livestock production systems. Livestock keeping is the predominant economic activity in the area, where the communities adopt seasonal mobility as a strategy for coping with seasonally available water and pasture resources. The total population of the Miyo and Moyale districts is 52,000 (50% female) and 31,000 (48% female), respectively (17). These areas were found in the southern arid and semi-arid parts of Ethiopia (18); a region that is highly vulnerable to climate change and recurring drought impacts resulting in widespread animal death, food insecurity, and conflicts. Moreover, population pressure, bush encroachment, and rangeland degradation are some of the added factors affecting the community. By comparison to Dugda and Humbo, they also suffer from poor access to health services and education, with few opportunities to engage in income-generating activities other than livestock (19).

### Data collection tool

The data collection tool covering a range of species and welfare topics was developed to collect relevant information to measure participants' KAP on animal welfare. The KAP questionnaire consists of a set of 34 items (questions) to determine knowledge (11 items), attitudes (10 items), and practice (13 items) among the respondents. The KAP questions covered a range of species and welfare topics including health (11) and nutrition (7), environment (2), behavioral (6) and mental/emotional state (8) dimensions of animal welfare. The responses of the items were measured on a Likert scale ranging from 1 to 5 (1 = strongly disagree to 5 = strongly agree) with higher scores indicating the most desired/undesired responses (Table 1). The socio-demographic characteristics of study participants such as age, gender, and occupation were also captured. While developing the tool, we reviewed different literature dealing with animal welfare and applied insights gained through community conversations with Ethiopian livestock owners from similar regions (12, 20). The questionnaire was then reviewed and assessed by subject experts and the research team for its content, design, validity, relevance, and understanding of the questionnaire items. Then, the questionnaire was pre-tested with farmers who were not included in the study population. The contents of the data collection tools were slightly modified based on the pilot survey, and suggestions from various people were included. The questionnaire was uploaded to a server for digital data collection using the open data kit (ODK) app installed onto tablets.

**Table 1:** Description of items used to assess the knowledge, attitude, and practice among livestock owners.

**Item code**	**Item description**	**Responses**
k	**Animal welfare knowledge**	1=strongly disagree, 2=disagree/disagree to some extent, 3=neutral (neither agree or disagree), 4=agree/agree to some extent, and 5=strongly agree
k1	Able to assess the amount and quality of feed	
k2	Free grazing is important for the animals	
k3	Animals need of sufficient, clean and comfortable area to lie down	
k4	Animals are sentient	
k5	Able to tell when animals are hungry or unhappy	
k6	Owner care affects how animals grow/produce	
k7	Bad handling leads to fear toward the owner	
k8	Untreated injuries affect the well-being and productivity of animals	
k9	Without enough water, animals' do not grow and produce milk	
k10	Animals can suffer from physical pain	
k11	I can quickly tell when one of my animals is sick	
at	**Animal welfare attitude**	
at1	I am confident in getting my animals to move where I want	
at2	My animals will learn more from being hit than instructed	
at3	Animals need to be able to perform their natural behaviors	
at4	I feel confident treating injuries that my animal may have	
at5	My animals must have enough water to drink	
at6	It is important to assess the health and welfare of my animals every day	
at7	I cannot influence how healthy my animals are	
at8	It is important to me that I care for my animals well	
at9	I believe my animals are happy and healthy	
at10	Animals need to feel safe in my care	
p	**Welfare practice scale**	
p1	My animals get enough to feed every day	
p2	I monitor the growth/weight of my animals	
p3	When I notice my animals are hungry, I act	
p4	My animals have a chance to move freely every day	
p5	I need to beat my animals to get them to do what I want	
p6	When I see an injury on my animal, I treat it	
p7	I consult with a trained health service provider when my animal is sick or injured	
p8	My animals can drink water whenever they want	
p9	It is common for my adult animals to get sick	
p11	My animals are exposed to heat or kept in poor housing.	
p12	Some of my animals suffer from lameness.	
p13	My animals walked long distances when selling and buying	

### Participants and data collection process

This KAP assessment was part of a larger baseline survey that was conducted to determine the welfare condition of the humans and animals in households across sites varying in agro-climatic zones and level of tree coverage. The information was collected from a total of 197 (106 men and 91 women) smallholder farmers across all the districts. The interviews were conducted in local languages by a trained expert from the National Agricultural Research System (NARS) from the respective study sites (21).

The study participant owned different animal species including cattle, sheep, goats, poultry, and donkeys, and camels were owned in pastoral households. The mean (median) herd/flock sizes owned by farmers included in the study ranged from 12.0 (median = 9) for cattle and 1 (median =1) for equine (Table 2).

**Table 2:** Mean (median) number of animal species owned by study participants according to production systems.

**Animal species**	**Production system**					
	**Mixed crop-livestock**		**Pastoral**		**Total**	
	**Mean**	**Median**	**Mean**	**Median**	**Mean**	**Median**
Cattle	8.2	7	14.6	11	12.0	9
Sheep	3.3	1	8.8	5	6.6	3
Goat	4.6	3	13.8	11	10.1	6
Equine	1.3	1	0.9	0	1.1	1
Poultry	5.8	5	2.6	1	3.9	2
Camel	.	.	2.4	0	2.4	0

### Ethical approval and consent from participants

This study received ethical approval from the International Livestock Research Institute Institutional Research Ethics Committee (ILRI IREC2020-43). The farmers/pastoralists were informed about the purpose of the study and the approximate time the interview will take, their right to withdraw at any time, and their anonymity and informed consent were obtained.

### Data analyses

Descriptive statistics were used to summarize the data. Items were measured on a Likert scale ranging from 1 to 5 (1 strongly disagree to 5 strongly agree). This scale was then recorded for analysis into a binary outcome (0/1) in which the correct or desirable responses were assigned a score of “1” and incorrect or undesirable responses were assigned “0.” Strongly agree with positive responses and strongly disagree with negative responses were categorized as desirable responses. For the attitude section, responses of “neither disagree nor agree” were excluded from the analysis, but this type of response was categorized as undesirable for knowledge and practice items. The item mean scores were transformed to a 0–100 scale for ease of interpretation. Unidimensionality of each scale, respectively knowledge, attitude, and practice, were determined using factor analysis assessing the size of eigenvalues, scree plots, and the magnitude of item loading from the first factor. The internal consistency of the scale was tested using Chronbach's alpha, to assess how good a scale is at measuring a concept. Chronbach's alpha ≥ 0.7 was considered to reflect good reliability of the scale (22, 23). Items for which a single underlying latent variable could not be measured were excluded from further analysis.

In Item response theory (IRT) modeling, the probability of a correct response to an item by an individual is assessed by the values of the latent variable (theta) and the characteristics of the item (24, 25). Two-parameter logistic regression IRT (2 PL) was fitted after confirming the unidimensionality assumption of the scale. Both an item's difficulty level and discrimination ability were evaluated. Item difficulty is also called item location parameter (b), which determined the 50 probabilities of responding correctly to a specific item given the respondent's ability. An item with a low level of difficulty (i.e., an easy item) was more likely to be answered correctly than an item with a high difficulty level. Item difficulty level between −4 and +4 was considered acceptable.

Item discrimination parameter (a), along with a plot of all item-specific information characteristic curves, allowed the determination of how well the items discriminate farmers/pastoralists with different levels of animal welfare knowledge, attitudes toward, and practices (16). The relationship between an individual's underlying trait and the probability of answering each question correctly was visualized using item characteristic curves (ICCs). Items with a ≤ 0.7 or excessively flat ICC curves were considered low discriminatory power and excluded from further analysis.

Item and test information function curves graphically depicted the amount of information each item and scale provided against a participant latent trait. The Item information function (IIF) for the 2pl model combined two-item parameters to indicate the amount of information provided by each item along with the θ value. The test characteristic curve (TCC) graph was plotted to show the expected scores from individuals with different latent trait levels. Scatterplots were added to TCC plots to assess the fit of expected scores with observed scores (26, 27).

Group IRT analyses were conducted to determine the probabilities of answering a given scale according to respondents' farming practices, gender, and tree access. Differential item functioning (DIF) analyses were performed to determine the likelihood of individual items responding differently with two groups (28, 29).

Mantel-Haenszel Tests (MH) were used to determine whether an item exhibited uniform DIF between the observed groups (farming practice, gender, tree access). That is, whether an item was answered in a “better” way by one group relative to the other for all values of the latent trait. Data analyses were carried out using STATA software program version 16 (Texas, USA).

## Results

### Demographic characteristics of the study participants

The demographic characteristics of the respondents are presented in Table 3. The mean age of participants was 42.5 (SD ± 15.3) years. Among the participants, 106 (53.8%) were men and 91 (46.2%) were women. Regarding respondents' main activities, 117 (59.4%) and 80 (40.6%) of them were pastoralists and mixed crop-livestock farmers, respectively.

**Table 3:** Sociodemographic characteristics of study participants in the pastoral and mixed crop-livestock production system.

**Categories**	**Production system, No (%)**		**Overall**
	**Pastoral**	**Mixed crop-livestock**	
Mean Age	42.6	42.3	42.5
Male	49 (41.9)	57 (71.3)	106 (53.8)
Female	68 (58.1)	23 (28.8)	91 (46.2)
Less tree accesses	69 (59.0)	40 (50.0)	109 (55.3)
Good tree accesses	48(41.0)	40 (50.0)	88 (44.7)

Values in the brackets represent the standard deviation.

### Livestock species and number owned

**Figure 2:**
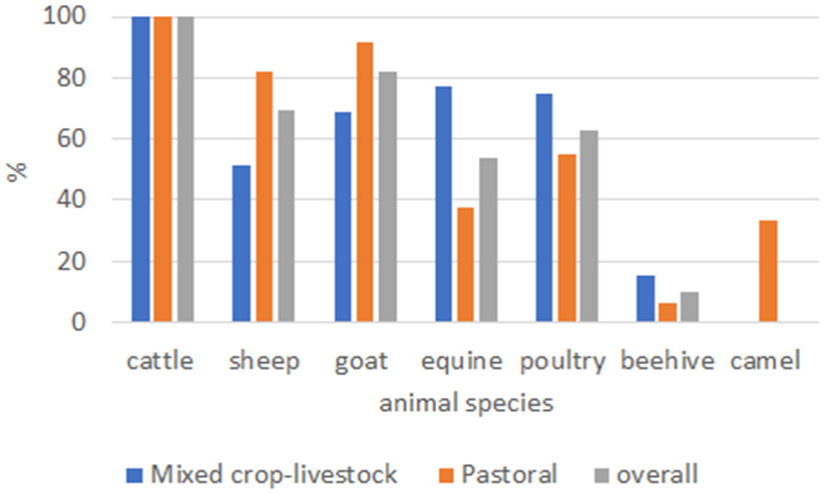
Livestock ownership status of respondents based on production system in Ethiopia.

Of the total interviewed households, 100% kept cattle, 69.5% kept sheep, 82.23% kept goats, 62.94% kept poultry, and 53.81% kept equine. Except for cattle, the ownership of other species significantly (*P* ≤ 0.05) varied between production systems (Figure 2). Additionally, 9.64% of the participants had beehives in their backyards. Regarding species diversity, the majority (63.5%) of the households owned more than three animal species on their farm.

### Psychometric properties of items and scales

From the factor analysis, all KAP scales were sufficiently unidimensional for the application of unidimensional IRT analysis and had good internal consistency reliability with Cronbach's α (Tables 4–**6**). Two items from the practice scale (p9 and p13) had loading below 3 and subsequently were not used in IRT parameter estimation. The discrimination (a) and difficulty (b) parameters from the IRT analysis of the KAP scale are presented in Tables 4–**6**, respectively. Item discrimination parameters ranged from 0.8 to 3.7 for knowledge, 1.0 to 2.1 for attitude, and 1.1 to 2.4 for practice scale. The difficulty parameters ranged from −0.2 to 0.8 for knowledge, and from 0.3 to 2.3 for the practice scale, suggesting that knowledge questions were easy to be answered correctly by at least 50% of respondents.

**Table 4:** Cronbach's alpha, IRT parameter estimates and uniform DIF for the animal welfare knowledge items.

**Item code**	**Item description**	**Cronbach's α**	***a***	***b***	**OR**	**95% CI**		***P*-value**
k1	Able to assess the amount and quality of feed	0.81	0.8	0.4	0.5	0.2	1.0	0.09
k2	Free grazing is important for the animals	0.81	0.8	0.5	0.2	0.1	0.5	0.00
k3	Animals need of sufficient, clean and comfortable area to lie down	0.80	1.3	0.2	0.6	0.3	1.3	0.26
k4	Animals are sentient	0.79	1.8	−0.2	0.4	0.1	0.9	0.04
k5	Able to tell when animals are hungry or unhappy	0.79	2.1	0.3	13.0	4.8	35.2	0.00
k6	Owner care affects how animals grow/produce	0.80	1.7	0.6	0.02	0.0	0.2	0.00
k7	Bad handling leads to fear toward the owner	0.80	1.7	0.8	3.3	1.5	7.5	0.00
k8	Untreated injuries affect the well-being and productivity of animals	0.78	3.7	0.1	0.8	0.3	2.0	0.75
k9	Without enough water, animals' do not grow and produce milk	0.78	3.4	−0.2	7.1	2.3	21.5	0.00
k10	Animals can suffer from physical pain	0.81	1.0	0.1	0.1	0.0	0.2	0.00
k11	I can quickly tell when one of my animals is sick	0.81	1.5	0.5	26.0	9.8	69.2	0.00
**k**	**Animal welfare knowledge scale**	**0.81**	**1.8**	**0.3**				

*a, discrimination parameter; b, difficulty parameter; OR, odds ratio; CI, confidence interval*. The bold values indicate the average value of the items or overall value of the knowledge, attitude and practice scales.

**Figure 3:**
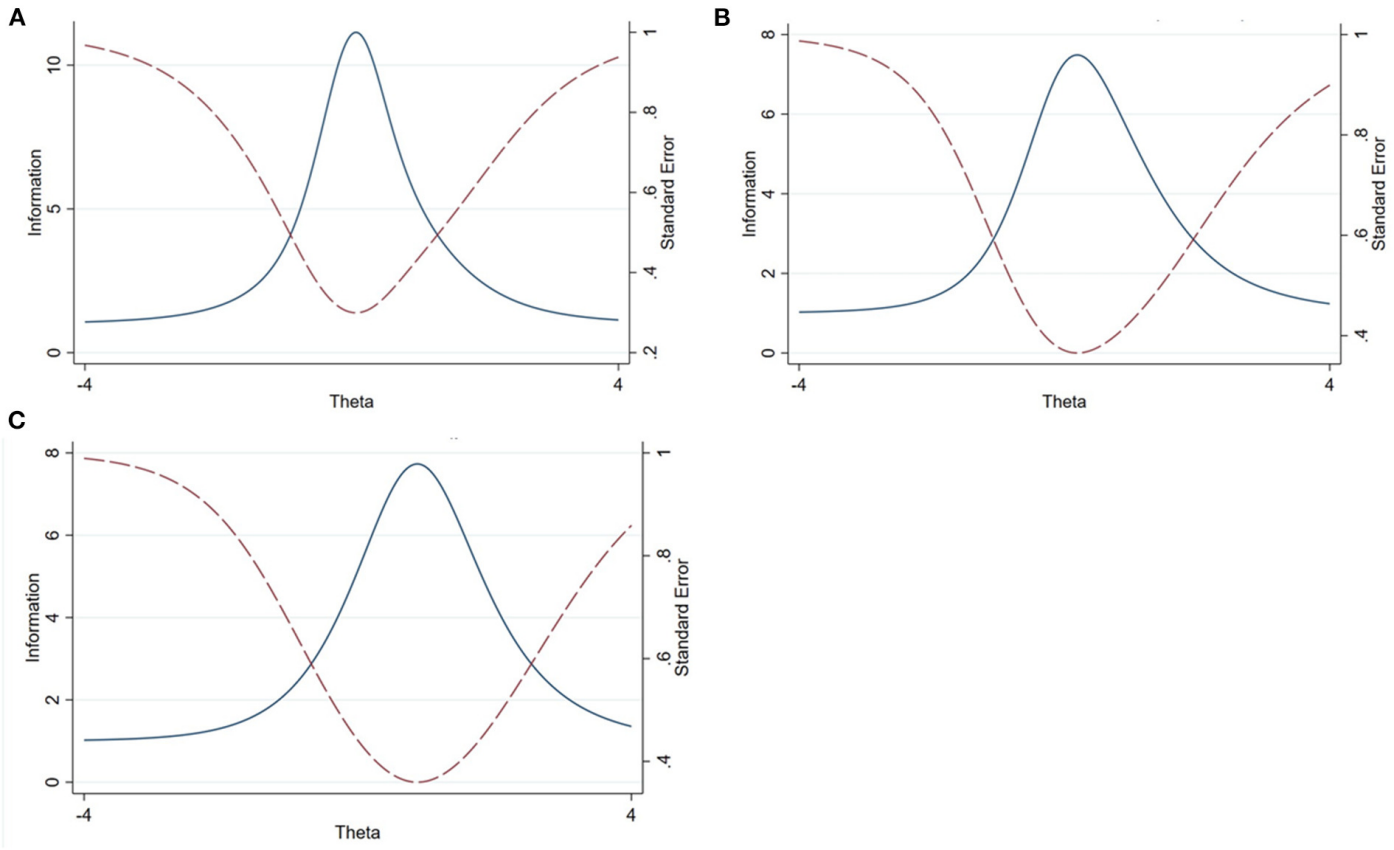
Test information function for animal welfare knowledge **(A)**, attitude **(B)**, and practice **(C)** scale. Blue line indicates “Test information” and red dot line indicate “Standard error.” The questions provided maximum information for respondents with knowledge level between −1 to 1.5, attitude level between −1 to 2 and practice level of −0.5 to 2.6.

**Figure 4:**
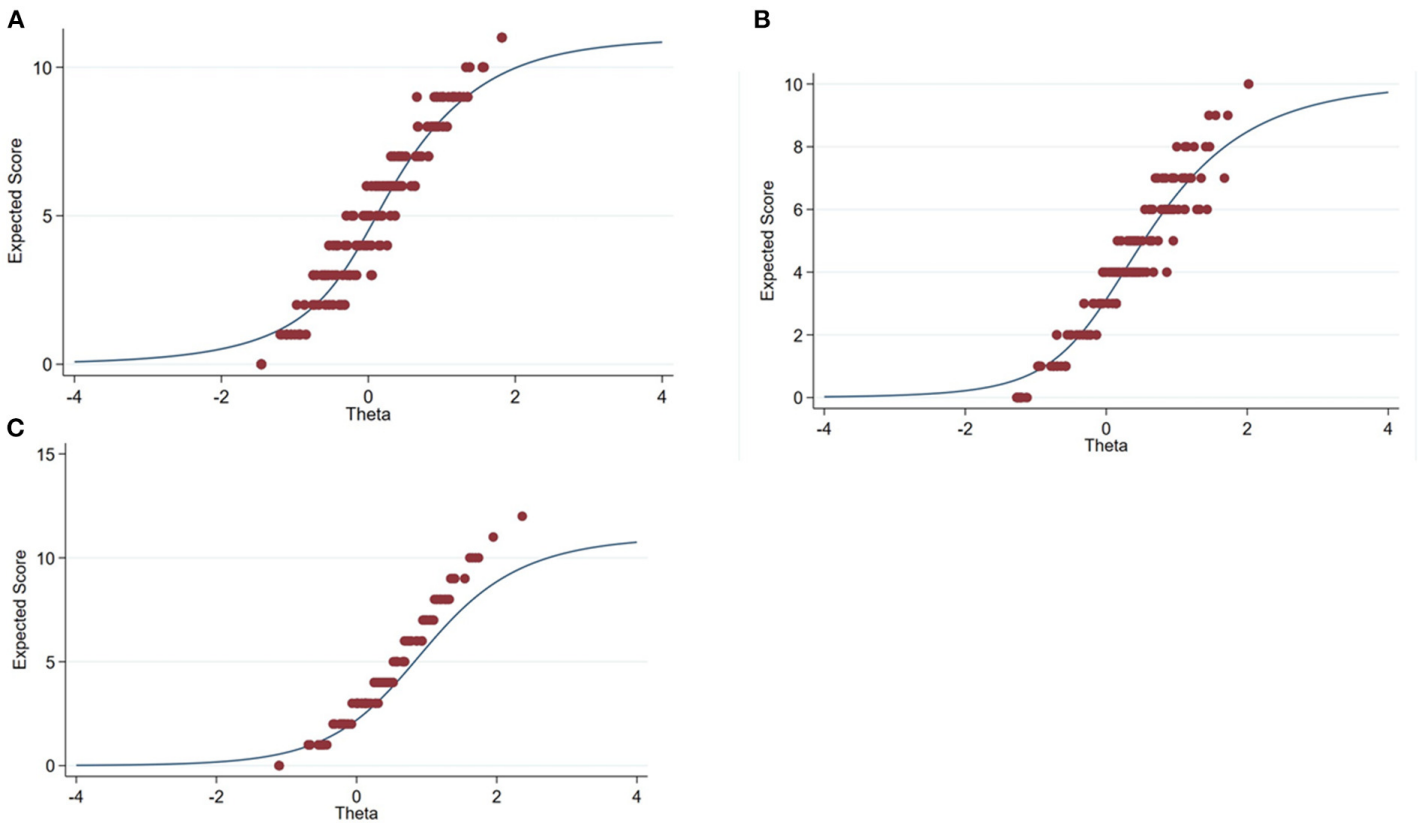
Test characteristic curve for animal welfare knowledge **(A)**, attitude **(B)**, and practice **(C)** with an added plot (red spots) of the summated score vs. ability (predicted score). Blue line indicates “expected score” and red dot indicate “total score.” Observed total score and expected score showed good fit for individual with knowledge between −0.8 and 1.4 and attitude level between −1 and 1.5 for the scale.

Test information functions of the KAP scales are displayed in Figure 3. The TCC plot shows the observed total score values vs. ability (expected score) overlaid (Figure 4). Evidence of good fit was observed for individuals with the latent trait between −0.8 and 1.4, for the knowledge scale, and between −1 and 1.5 for the attitude scale. However, the observed total score shows evidence of deviation from the expected score, particularly for individuals with a latent practice level between −0.6 and 0.5 on the practice scale.

### Animal welfare knowledge

**Figure 5:**
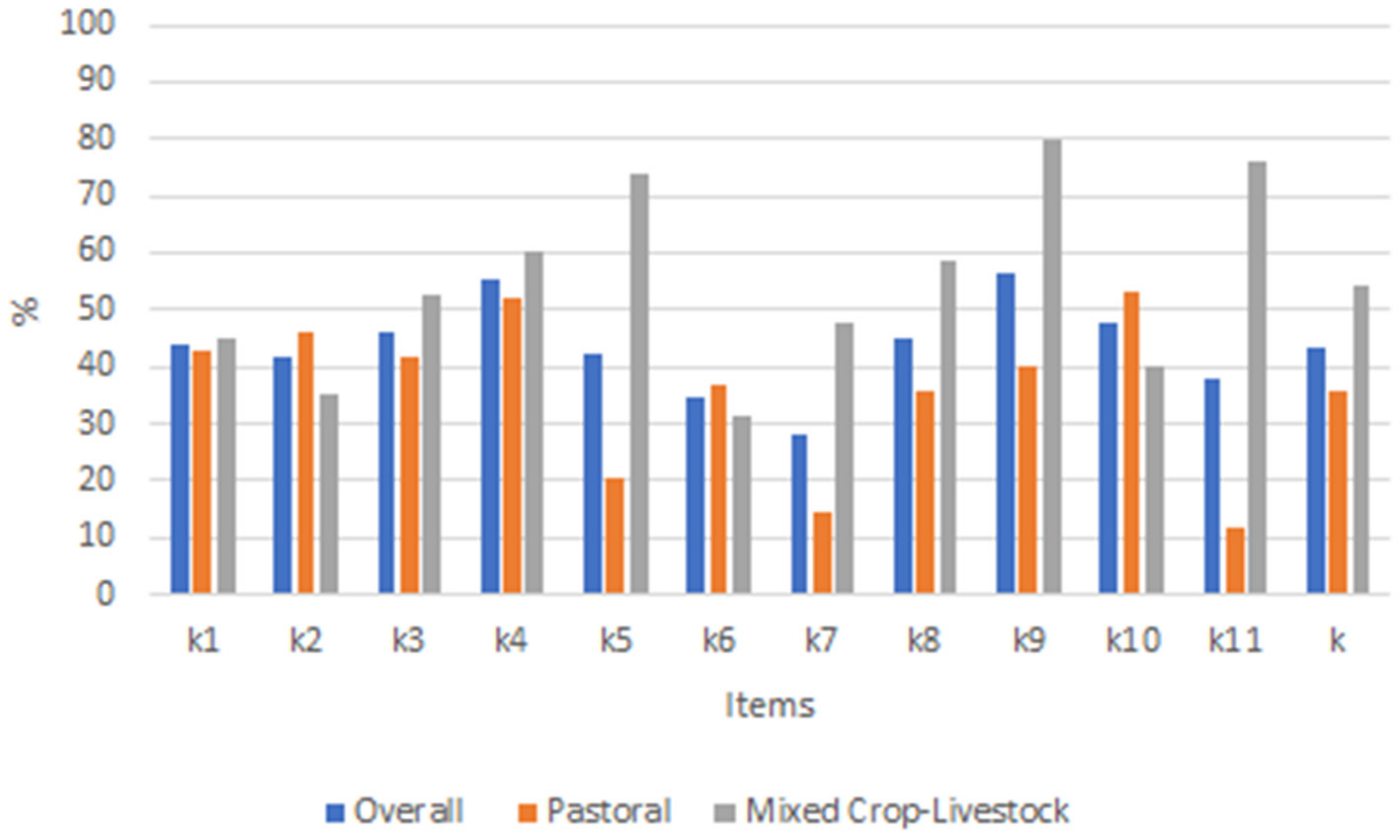
Percent of correct responses for animal welfare knowledge items aggregated by production system in Ethiopia.

The total knowledge score ranged from 0 (incorrect) to 11 (all correct) and the mean (±SD) score was 4.8 (±3.2). The list of all items, along with the percentage of correct answers, aggregated by the production system is shown in Figure 5. Overall, the percentage of correct responses was 43.5%. Mixed crop-livestock farmers answered more correct responses than pastoralists (54.5 vs. 36.0%). The mean percent of correct responses was similar for male (43.4%) and female (43.6%) respondents (Supplementary Table 1). The respondents recorded the lowest score for item k7 (27.91%) which related to the animal-human relationship (“bad handling leads to fear toward the owner”) and the highest score (56.3%) for item k9 related to the biological needs of the animal (“without enough water, animals” do not grow and produce milk'). The desired percent of correct knowledge, i.e., an average of responses above 50%, was recorded for two statements only (item k4 “Animals are sentient” and k9) (Figure 5).

Group IRT analysis result showed that mixed crop-livestock farmers had better knowledge of animal welfare than pastoralists with a mean θ value of 1.2 (95% CI: 0.7, 1.7) and a variance of θ of 1.9 (95% CI: 1, 3.89), which had expected values 0 and 1, respectively. The difference is statistically significant (*p* = 0.00). However, there was no significant knowledge difference between male and women livestock keepers (*p* = 0.91) with a mean θ value of −0.02 (95% CI: −0.33, 0.29) and a variance of θ of 1.05 (95% CI: 0.57, 1.98).

The MH DIF test result showed that all items in the knowledge scale demonstrated significant differential item functioning, except for three items related to animal feed resource (k1), housing (k3), and wound management (k8) (Table 4). However, none of the items showed DIF related to gender and tree access. Mixed crop-livestock farmers have better knowledge of items related nutrition condition of the animal (k5), the animal-human relationship (k7), the importance of water for growth and milk production (k9), and health inspection (k11) than pastoralists. Pastoralists have better knowledge of items related to natural behavior expression (k2), animal care (k6), and animal suffering (k10) than mixed crop-livestock farmers. Likelihoods of mixed crop-livestock farmers to respond correctly to items k5, k7, k9, and k11 correctly were 13, 3.3, 7.1, and 26-times higher than that of pastoralists, respectively. Nevertheless, the pastoralist had 50, 5, and 10-times higher odds to respond to items k2, k6, and k10 correctly, respectively.

### Animal welfare attitude

**Figure 6:**
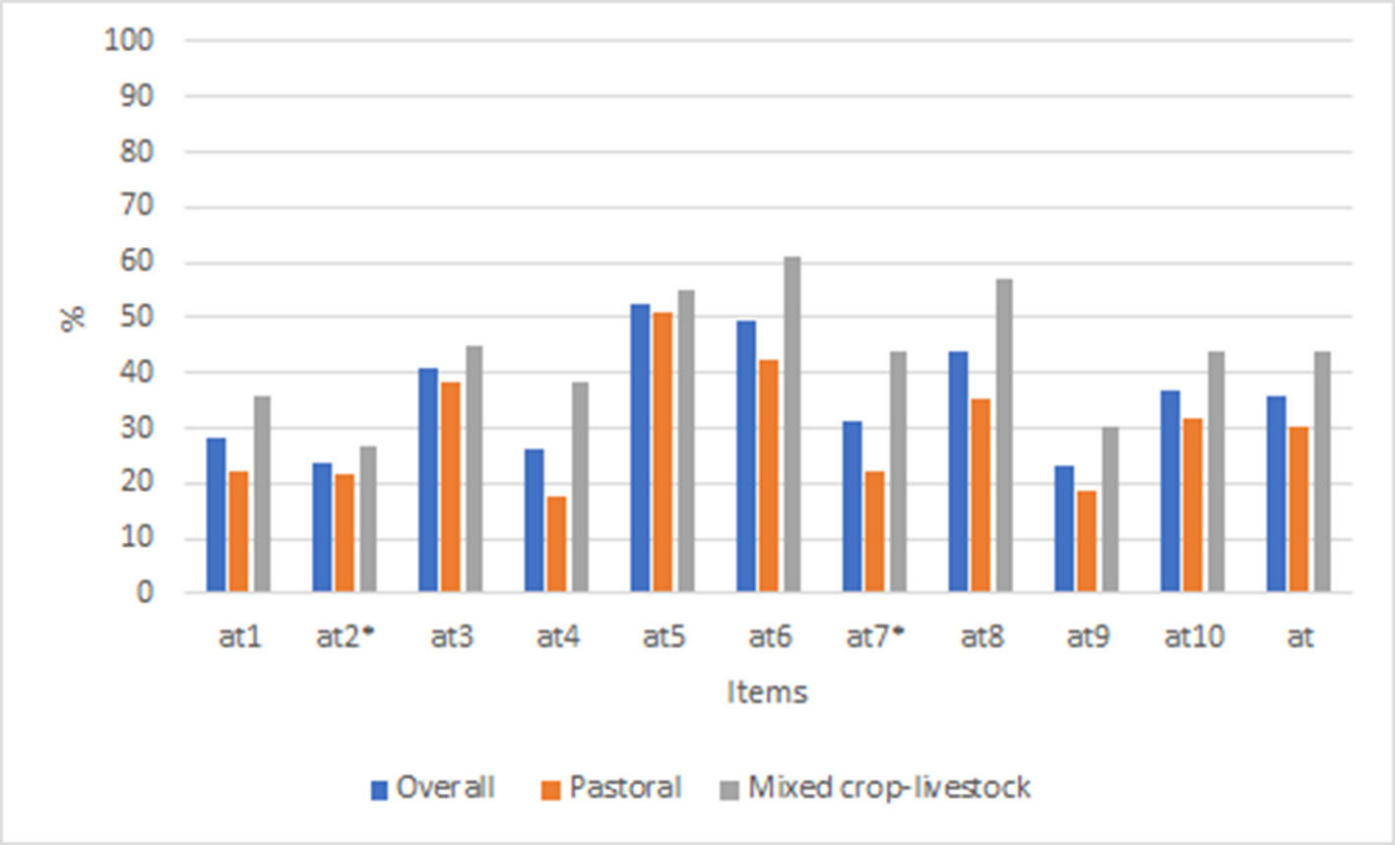
Percent of correct responses for animal welfare attitude items aggregated by production system in Ethiopia. Items with “*” indicate the scale were reversed.

From the total of 10 points, the mean (±SD) score of desirable attitudes was 3.4 (±0.2). The list of all questions, along with the percent of desired responses aggregated by the livestock production system, is shown in Figure 6. Overall, the percentage of correct responses for the attitude scale was 35.7%. Mixed-crop livestock farmers answered more questions “correctly” than pastoralists (43.7 vs. 30.1%). A slightly higher mean percent of desired responses were obtained for female (37.3%) than male (34.3%) respondents (Supplementary Table 1). The percent of desired responses for the individual question ranged from 23.4% for item at9 (“my animals are happy and healthy”) to 52.6% for item at5 (“my animals must have enough water to drink”). The respondents scored above 50% desired response for only one statement (item at5).

The group IRT analysis result showed that mixed crop-livestock farmers had a better attitude toward animal welfare than pastoralists (*p* = 0.002) with a mean θ value of 0.54 (95% CI: 0.2–0.9) and variance of θ of 1.1 (95% CI: 0.5–2.1). Nevertheless, attitudes toward animal welfare did not show significant differences between men and women and respondents with good and less tree access.

From the MH DIF test result, only one item from the attitude scale, at2 (“my animals will learn more from being hit than instructed”) had significant differential item functioning related to the production system (Table 5), and none of the items had DIF related to tree access and gender. Pastoralists had 3.3-times higher odds than mixed crop-livestock farmers to have a positive attitude about how to train their animals (OR = 0.3, 95% CI = 0.1–0.9, *p* = 0.05).

**Table 5:** Cronbach's alpha, IRT parameter estimates, and uniform DIF for animal welfare attitude items.

**Item code**	**Items descriptions**	**Cronbach's α**	***a***	***b***	**OR**	**95%CI**		***P*-value**
at1	I am confident in getting my animals to move where I want	0.76	1.62	0.9	1.3	0.5	3.3	0.80
at2*	My animals will learn more from being hit than instructed	0.78	0.86	1.5	0.3	0.1	0.9	0.05
at3	Animals need to be able to perform their natural behaviors	0.75	1.89	0.3	0.6	0.2	1.6	0.40
at4	I feel confident treating injuries that my animal may have	0.77	1.52	0.9	2.6	0.9	7.7	0.13
at5	My animals must have enough water to drink	0.75	2.41	0.0	1.0	0.4	2.6	0.85
at6	It is important to assess the health and welfare of my animals every day	0.75	2.37	0.0	1.6	0.7	4.0	0.41
at7*	I cannot influence how healthy my animals are	0.77	0.99	0.9	0.9	0.4	2.2	0.99
at8	It is important to me that I care for my animals well	0.75	1.84	0.2	2.2	0.8	5.9	0.19
at9	I believe my animals are happy and healthy	0.78	0.91	1.5	0.7	0.2	2.0	0.64
at10	Animals need to feel safe in my care	0.76	1.66	0.5	0.7	0.2	2.0	0.67
**at**	**Animal welfare attitude**	**0.78**	**1.61**	**0.7**				

^*^Scale reversed; a, discrimination parameter; b, difficulty parameter; OR, odds ratio; CI, confidence interval. The bold values indicate the average value of the items or overall value of the knowledge, attitude and practice scales.

### Animal welfare practices

**Figure 7:**
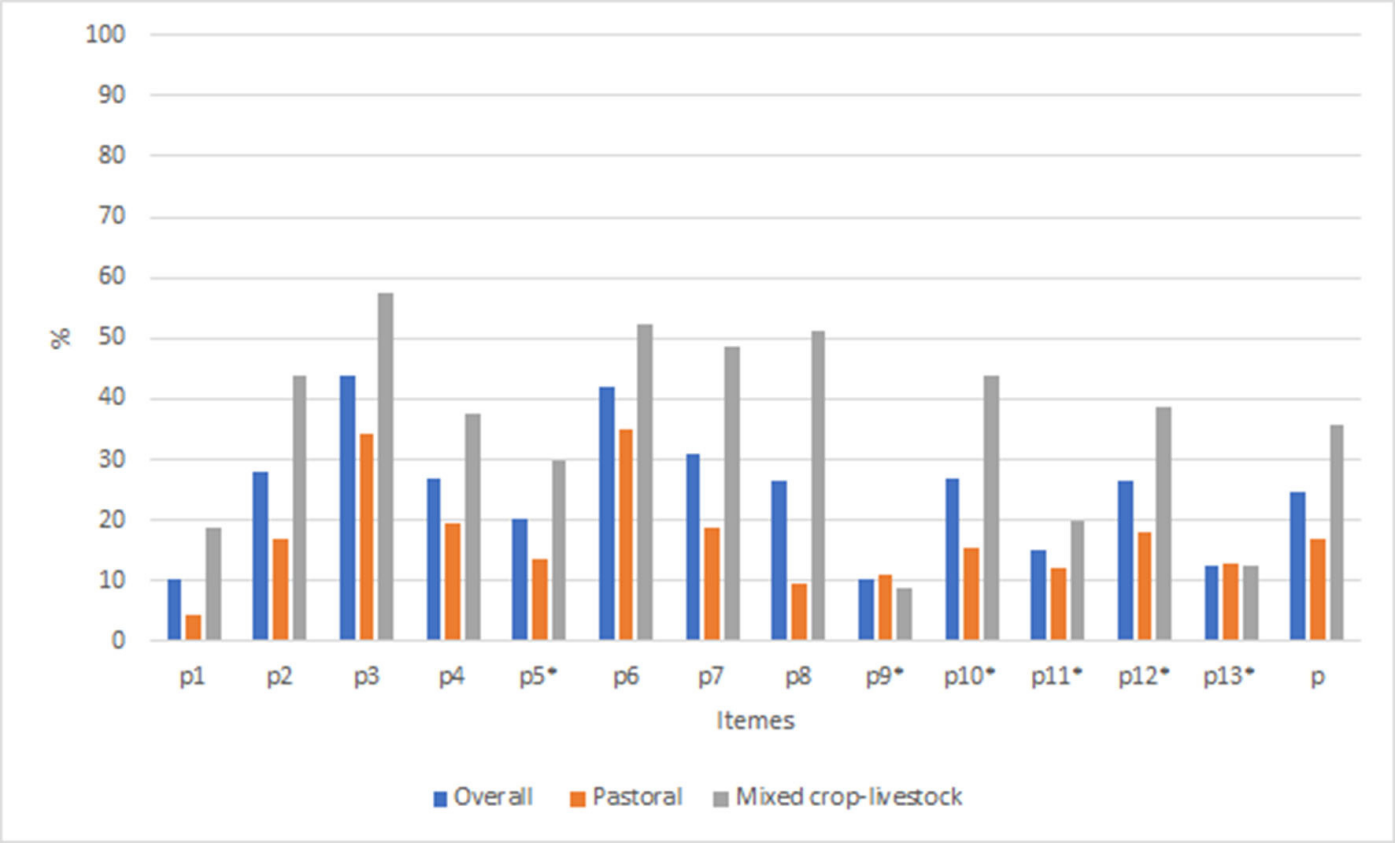
Percent of correct responses for animal welfare practice items aggregated by production system in Ethiopia. Items with “*” indicate the scale were reversed.

From a total of 13 points, the mean (±SD) score of correct practice was 3.2 (± 0.2). The list of all practice questions, along with the percent of correct responses aggregated by the livestock production system, is shown in Figure 7. Overall, the mean percent of correct responses for self-reported practice was 26.4%. A slightly higher mean percentage of correct responses was obtained for male (26.8%) than for female (22.1%) respondents (Supplementary Table 1). The mean correct response ranged from 10.2% for item p1 (“my animals get enough to feed every day”) to 43.7% for item p3 (“when I notice my animals are hungry, I act”) for individual items. The respondents scored all the statements below the required average (50%) animal practice level.

The group IRT analysis result showed mixed crop-livestock farmers had better self-reported animal welfare practices than pastoralists (*p* = 0.00), with a mean θ value of 1.2 (95% CI: 0.8–1.5) and variance of θ of 0.7 (95% CI: 0.3–1.5).

The result of the MH DIF test for practice scale items showed that only one item, p8 (“my animals can drink water whenever they want”) had significant differential item functioning related to the production system (Table 6) and none of the items had DIF related to tree access and gender. Mixed crop-livestock farmers had a 3.4 times higher probability to provide water for their animals whenever they want than pastoralists (OR = 3.4, 95% CI = 1.3–8.9, *p* = 0.01).

**Table 6:** Cronbach's alpha, IRT parameter estimates, and uniform DIF for animal welfare practice items.

**Item code**	**Item description**	**Cronbach's α**	***a***	***b***	**OR**	**95% CI**		***P*-value**
p1	My animals get enough to feed every day	0.8	1.2	2.3	2.4	0.6	8.6	0.28
p2	I monitor the growth/weight of my animals	0.8	1.6	0.9	1.2	0.5	2.7	0.87
p3	When I notice my animals are hungry, I act	0.8	1.5	0.3	0.8	0.4	1.9	0.80
p4	My animals have a chance to move freely every day	0.8	1.5	1.0	0.6	0.2	1.5	0.40
p5*	I need to beat my animals to get them to do what I want	0.8	2.1	1.1	0.5	0.2	1.6	0.34
p6	When I see an injury on my animal, I treat it	0.8	1.1	0.4	0.7	0.3	1.4	0.39
p7	I consult with a trained health service provider when my animal is sick or injured	0.8	1.6	0.7	1.4	0.6	3.3	0.62
p8	My animals can drink water whenever they want	0.7	2.3	0.8	3.4	1.3	8.9	0.01
p9*	It is common for my adult animals to get sick	0.7	0.9	2.7	0.1	0.0	0.6	0.00
p10*	When an animal is sick, I cannot influence its recovery	0.7	1.9	0.8	1.2	0.5	2.9	0.81
p11*	My animals are exposed to heat or kept in poor housing.	0.8	1.7	1.5	0.3	0.1	1.1	0.10
p12*	Some of my animals suffer from lameness.	0.8	1.1	1.1	1.1	0.5	2.5	0.95
p13*	My animals walked long distances when selling and buying	0.7	1.1	2.1	0.3	0.1	1.0	0.07
***p***	**Welfare practice scale**	**0.8**	**1.5**	**1.2**				

*Scale reversed; a, discrimination parameter; b, difficulty parameter; OR, odds ratio; CI, confidence interval. The bold values indicate the average value of the items or overall value of the knowledge, attitude and practice scales.

### Correlation between respondents' knowledge, attitude, and practice

**Figure 8:**
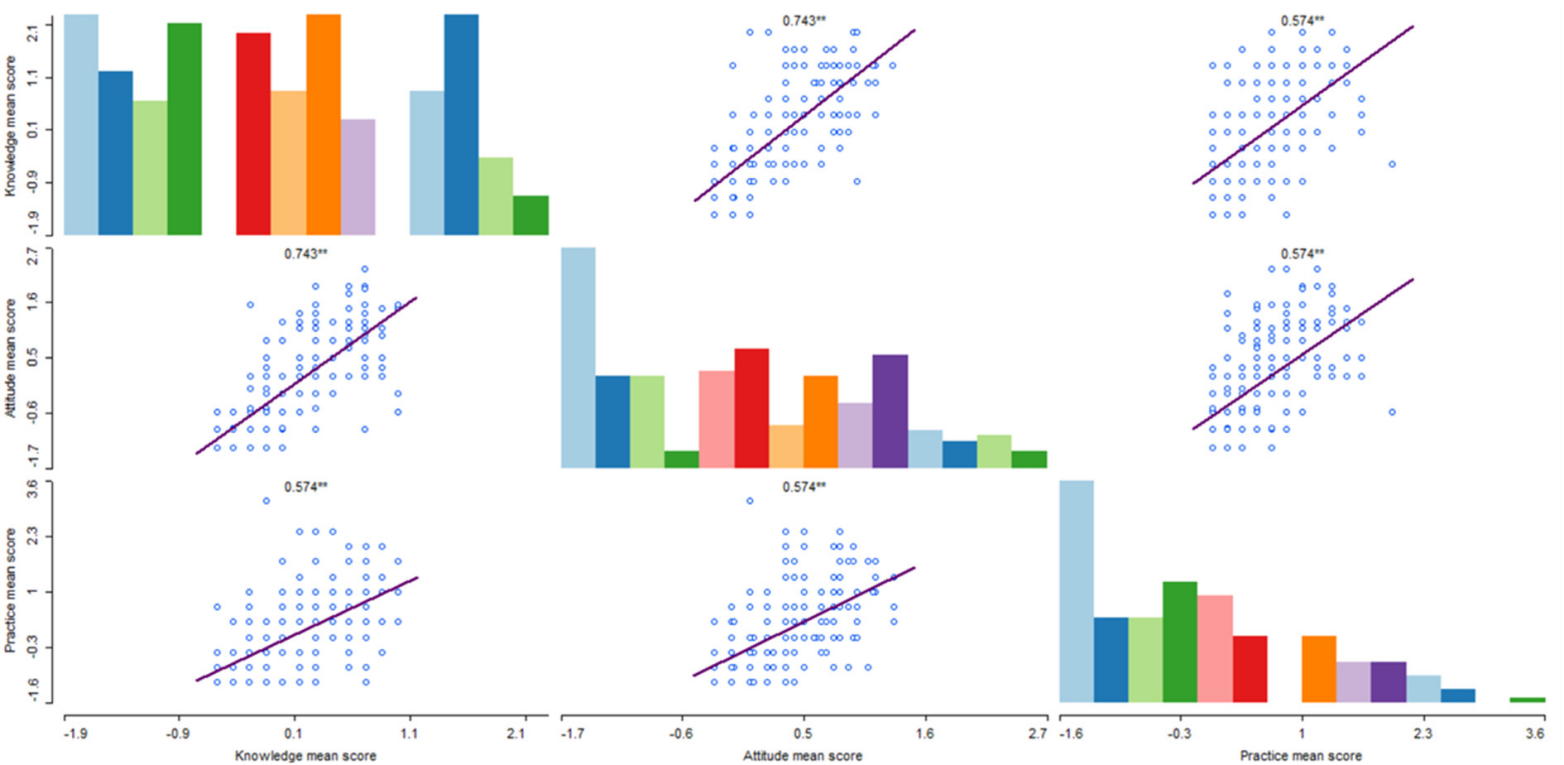
Correlation matrix which show the relationship of KAP score among smallholder farmers in Ethiopia.

A Pearson correlation analysis was conducted to assess the relationship between the total score of the KAP scales. Figure 8 shows the relationship between knowledge, attitude, and practice. There was a significant positive association between respondents' knowledge and attitude toward animal welfare (*r* = 0.74, *p* = 0.00), suggesting having appropriate knowledge explains 54.8% of the positive attitude the respondents developed. Similarly, there was a strong positive association between respondents' knowledge and self-reported practice (*r* = 0.57, *p* = 0.00), suggesting having appropriate knowledge explains 32.5% of good animal welfare practices. Good practices also had a strong and positive correlation with desirable attitudes (*r* = 0.57, *p* = 0.00), having a desirable attitude explaining 32.5% of good animal welfare practices.

## Discussion

This study provided a summary of animal welfare KAP results and evaluate the reliability of the assessment tools in three communities in Ethiopia. The finding showed that a higher score was recorded for the animal welfare knowledge scale followed by attitude. However, overall, the livestock owners had inadequate knowledge of animal welfare, undesirable attitude toward the animals they handle, and suboptimal animal welfare practices.

Animal welfare KAP from across Africa are limited. Another study has documented a lack of deep knowledge of most of the critical animal welfare issues, undesirable attitudes, and poor welfare practices among stock persons in Kenya (15). The roles of animal owners to abattoir stock people are markedly different, in terms of responsibility, ownership, and connection to animals. The poor attitude and practices toward animal welfare recorded in this study might be related to inadequate knowledge, which might relate to low awareness of the farming community on the physical, biological, and behavioral requirements of the animals. A lack of appropriate information on animal welfare may prevent owners from developing a positive attitude toward animal welfare (30–32) and as a result, fail to improve practice (33, 34). Access to animal welfare-related information and training initiatives to improve livestock welfare is considered important to increase the awareness of the farmers on animal welfare (4, 35), and seems to be lacking in the Ethiopian agricultural extension system (36).

The result of this study showed that mixed crop-livestock farmers had a better KAP score than pastoralists. Mixed crop-livestock farmers have better access to extension and veterinary services which enable them to have a better awareness of animal care and management and implement animal health-related activities than pastoralists (37). This may in part explain the geographical differences. Pastoralists are mobile with their livestock and move in response (at least in part) to the availability of feed and water resources. This movement process can hamper pastoralist access to information and basic animal health care and extension services (38, 39). Public-private partnership (PPP) model which creates enabling environments for efficient use of available resources or to expand coverage of veterinary health services (40, 41) is one approach that can be promoted in pastoral areas to address challenges in animal health and could include animal welfare perspectives as part of the PPP contract.

The difference in the perceptions of the farmers on animal welfare is influenced by geographical, economic, social and environmental and cultural, and religious beliefs, and may often be different from the welfare needs of the animal (6, 14, 42). This may further, in part, explain some differences in crop-livestock and pastoralist KAP score. Community members attach different values or meanings to animals depending on the purpose of the animals and their relationships with the animals. For instance, women value and have a closer relationship with dairy cows, while men focus on social status and prestige, and thus attach more value to cattle and their number (12). Mixed crop farmers have frequent interaction with their animals due to the smaller herd size and the use of animals for crop agriculture and transport (5). The pastoralists have intimate knowledge and connection with their animals, and the animals in the pastoral production system tend to move freely within the rangeland in the search of feed and water and exhibit their natural behaviors without restriction (43, 44). These different roles that animals play in the two different agricultural systems relate to the difference in responses seen in the current paper.

The prevalence of poor practices recorded in this study related to animal feed needs enormous improvement. Under an Ethiopian extensive production system, the livestock often spends the whole day without enough feed and water (45, 46). Moreover, pastoral production systems are practiced in drylands agroecosystems where multiple stressors such as excessive heat, and the need to walk long distances to source feed and water create further welfare compromises for the animal (47). Feed and water resource improvement strategies, such as silvopastoral or agropastoral farming systems, have been demonstrated to have a positive impact on animal welfare (48, 49). These systems can be promoted and adopted across both pastoralist-dominated and crop-livestock landscapes. Agro-ecologically apprapriate tree presence in both crop-livestock and pastoral systems is likely to have an encouraging influence on animal welfare and productivity, particularly by allowing the expression of natural behavior, and providing shade and quality feeds (49, 50).

Inappropriate management practices, such as beating, were both highlighted in this study and have been previously described in similar settings, especially in the mixed crop-livestock production system (5, 42). Actions to improve the empathy of owners toward their animals, and encourage low-stress handling practices, could further help to improve animal welfare.

All knowledge, attitude, and practice set of questions used in this study met the unidimensional assumption of the IRT model. It showed good reliability with acceptable Cronbach's alpha value and fit well with the scale. From the parameters estimate, the knowledge scale had a higher discrimination ability than the attitude and practice scales. The statements in the knowledge scale were relatively easy for the respondents with higher probabilities of responding to them correctly. This implies the livestock owners had better animal welfare knowledge than a positive attitude and good practice which might be acquired through experience, training, from opinion leaders, and peer-to-peer learning. It also likely reflects the barriers that owners face to address these known animal welfare needs. For example, an animal owner can know the correct answer to K9 “Without enough water, animals” do not grow and produce milk', but be unable to turn it into practice (i.e., P8 “My animals can drink water whenever they want”).

This is important to consider when turning the results from the current study into practice change to improve animal welfare. Community-based engagement and learning processes, called Community Conversations have the potential to both increase awareness of an issue and generate community-led steps to address issues (12). Community Conversations collectively identify community strengths, knowledge gaps, and constraints, analyze community values and practices, and explore strategies for addressing challenges (20). This approach can make Community Conversations a particularly useful tool for animal welfare improvements because it creates awareness of issues and enables the participants to then create solutions. For example, participants can become more aware of issues with poor animal handling and limited water availability, and then pledge to take a gentler approach to handle and build community troughs to improve water access for animals while grazing.

This study was not without limitations. The study participants were selected purposively based on their tree access which makes the results difficult to generalize to other small holders farmers across the vast agroecology and production systems of Ethiopia. Future studies with randomly selected participants across different agroecology and production system of Ethiopia should be conducted. A single-visit self-report interview approach may lead to a concept called social desirability bias (51) where some of the study participants describe actions that do not always reflect their actual practices. Further studies which longitudinally measure livestock owners' routine animal management practices and their impact on the welfare indicators of their animals should be considered.

## Conclusion

This study found a positive correlation between the knowledge of the farmers and their attitude toward animal welfare and self-reported practices. This implied positive attitude and good animal welfare practice can be achieved through appropriate training which improves the awareness of the farmers on the biological, physical, and mental needs of their animals. The livestock production system influenced livestock keeper's animal welfare KAP, and it is likely that this is related to resource availability, and potentially due to different approaches in livestock ownership. The developed questionnaire had satisfactory psychometric properties in terms of measuring animal welfare KAP in Ethiopian smallholder farmers, making it suitable for the measurement of the impact of the intervention on animal welfare. It is also recognized that the ability to intervene to improve animal welfare may be limited, depending on the owners' production system and resources.

## Data availability statement

The raw data supporting the conclusions of this article will be made available by the authors, without undue reservation.

## Author contributions

RD, BW, TB, and MM conceived the study. GA and TB implemented the study. GA, EG, TB, JJ, and JM followed up and monitored data collection. TK-J supervise the overall implementation of the project activities. GA analyzed the data and prepared the first draft of the manuscript with RD. All authors made contributions to the conception, design, and revision of the manuscript. All authors reviewed and approved the final version.

## Funding

This study was funded by the Biovision Foundation for Ecological Development (Grant Nos. BV DPA_009/2020-2021 and BV DPA_012 2021) and received support from the CGIAR Research Program on Livestock and HEAL project. The funders played no role in the design or conclusion of the study.

## Conflict of interest

The authors declare that the research was conducted in the absence of any commercial or financial relationships that could be construed as a potential conflict of interest.

## Publisher's note

All claims expressed in this article are solely those of the authors and do not necessarily represent those of their affiliated organizations, or those of the publisher, the editors and the reviewers. Any product that may be evaluated in this article, or claim that may be made by its manufacturer, is not guaranteed or endorsed by the publisher.
