# Supplementary material for: Animal welfare knowledge, attitudes, and practices among livestock holders in Ethiopia
Source: Front Vet Sci. 2022 Nov 7;9:1006505. doi: 10.3389/fvets.2022.1006505 (PMC9676930; doi:10.3389/fvets.2022.1006505)
Supplement: Supplementary file 1 [file Table_1.docx]

Table 1 Percent of correct responses for animal welfare knowledge, attitude and practice items aggregated by respondents’ gender

| Items | items description | % Of correct practice | | |
| --- | --- | --- | --- | --- |
|  |  | Male | Female | Overall |
| **k** | **Animal welfare knowledge scale** | 43.4 | 43.7 | 43.5 |
| k1 | Able to assess the amount and quality of feed | 46.2 | 40.7 | 43.7 |
| k2 | Free grazing is important for the animals | 38.7 | 45.1 | 41.6 |
| k3 | Animals need of sufﬁcient, clean and comfortable area to lie down | 48.1 | 44.0 | 46.2 |
| k4 | Animals are sentient | 52.8 | 58.2 | 55.3 |
| k5 | Able to tell when animals are hungry or unhappy | 42.5 | 41.8 | 42.1 |
| k6 | Owner care affects how animals grow/produce | 27.4 | 42.9 | 34.5 |
| k7 | Bad handling leads to fear of the owner | 31.1 | 24.2 | 27.9 |
| k8 | Untreated injuries affect the well-being and productivity of animals | 44.3 | 46.2 | 45.2 |
| k9 | Without enough water, animals’ do not grow and produce milk | 59.4 | 52.7 | 56.3 |
| k10 | Animals can suffer from physical pain | 45.3 | 50.5 | 47.7 |
| k11 | I can quickly tell when one of my animals is sick | 41.5 | 34.1 | 38.1 |
| **at** | **Animal welfare attitude** | **34.3** | **37.3** | **35.7** |
| at1 | I am confident in getting my animals to move where I want | 27.7 | 28.8 | 28.2 |
| at2* | My animals will learn more from being hit than instructed | 22.7 | 25.3 | 23.9 |
| at3 | Animals need to be able to perform their natural behaviors | 35.2 | 47.8 | 41.0 |
| at4 | I feel confident treating injuries that my animal may have | 26.5 | 26.0 | 26.3 |
| at5 | My animals must have enough water to drink | 52.8 | 52.2 | 52.6 |
| at6 | It is important to assess the health and welfare of my animals every day | 49.5 | 50.0 | 49.7 |
| at7* | I cannot influence how healthy my animals are | 27.5 | 35.6 | 31.2 |
| at8 | It is important to me that I care for my animals well | 44.2 | 43.8 | 44.0 |
| at9 | I believe my animals are happy and healthy | 22.8 | 24.1 | 23.4 |
| at10 | Animals need to feel safe in my care | 34.3 | 39.8 | 36.8 |
| **p** | **Welfare practice scale** | **26.8** | **22.1** | **24.6** |
| p1 | My animals get enough to feed every day | 12.3 | 7.7 | 10.2 |
| p2 | I monitor the growth/weight of my animals | 31.1 | 24.2 | 27.9 |
| p3 | When I notice my animals are hungry, I act | 48.1 | 38.5 | 43.7 |
| p4 | My animals have a chance to move freely every day | 27.4 | 26.4 | 26.9 |
| p5* | I need to beat my animals to get them to do what I want | 20.8 | 19.8 | 20.3 |
| p6 | When I see an injury on my animal, I treat it | 42.5 | 41.8 | 42.1 |
| p7 | I consult with a trained health service provider when my animal is sick or injured | 32.1 | 29.7 | 31.0 |
| p8 | My animals can drink water whenever they want | 33.0 | 18.7 | 26.4 |
| p9* | It is common for my adult animals to get sick | 14.2 | 5.5 | 10.2 |
| p10* | When an animal is sick, I cannot influence its recovery | 29.2 | 24.2 | 26.9 |
| p11* | My animals are exposed to heat or kept in poor housing. | 17.0 | 13.2 | 15.2 |
| p12* | Some of my animals suffer from lameness. | 29.2 | 23.1 | 26.4 |
| p13* | My animals walked long distances when selling and buying | 11.3 | 14.3 | 12.7 |

Items with * indicate the scale were reversed
